# Teratogenic Effects of Pregabalin in Mice 

**Published:** 2013-10

**Authors:** Leila Etemad, Afshar Mohammad, Amir Hooshang Mohammadpour, Nasser Vahdati Mashhadi, Seyed Adel Moallem

**Affiliations:** 1 Pharmaceutical Research Center, Mashhad University of Medical Sciences, Mashhad, Iran; 2 Department of Anatomy, Birjand University of Medical Sciences, Birjand, Iran; 3 Medical Toxicology Research Center, Mashhad University of Medical Sciences, Mashhad, Iran; 4 Departments of Pharmacodynamics and Toxicology, School of Pharmacy, Mashhad University of Medical Sciences, Mashhad, Iran

**Keywords:** Antiepileptic drugs, Developmental toxicity, Homocysteine, Teratogenicity

## Abstract

***Objective(s):*** Anti-epileptic drugs (AEDs) have the potential to affect fetal development throughout pregnancy. Considering the broad therapeutic indications of pregabalin (PGB), its potential teratogenic effects and the levels of homocysteine have been studied.

***Materials and Methods:*** Timed-pregnant mice received one of three doses of PGB (20, 40 or 80 mg/kg/day) or the vehicle control during organogenesis, intraperitoneally. The litters were stained and examined for malformations. Total homocysteine (tHcy) was measured in serum from the pregnant mice on GD18.

***Results:*** The rate of fetus malformations increased significantly in all treated groups as compared to the control group. The abnormalities included limb, vertebral column and craniofacial abnormalities. The most common abnormality was limb deformity. The percentage of fetal resorption significantly increased at higher doses. There was no significant difference in tHcy concentrations between the treated and control groups.

***Conclusion:*** Pregabalin may have potential teratogenic effects even in lower doses, however with less intensity than other AEDs. Therefore, it is suggested that great caution should be taken when prescribing it in pregnancy and further investigation for possible mechaninsms.

## Introduction

Drug therapy is an important challenge during pregnancy, especially in patients involved in long lasting diseases. Due to relapsing and severity of underlying diseases, many women should not stop using medications (1). Epilepsy is a chronic and disabling neurologic disorder. Approximately half of epileptic patients are women (2, 3). Currently, management of epilepsy is mainly based on antiepileptic drugs (AEDs). Therefore the total number of children exposed to epileptic drugs is considerably high (4).

AEDs have the potential to affect fetal development throughout pregnancy. Although the majority of children born to women with epilepsy are normal, they are at increased risk for malformations (5). Several studies show that AED therapy rather than the maternal disease or convulsions are the cause of malformations identified at birth. Annergers and colleagues found that the rates of malformation in the offspring of epileptic mothers treated with AEDs are higher than in the children of normal group (6).

Use of traditional antiepileptic drugs (e.g., phenobarbital, phenytoin, carbamezapine, valproate) during pregnancy is correlated with increased risk of major congenital malformations (7, 8). Thus, finding new safer drugs for mother and fetus seems to be necessary. It is asserted that newer AEDs leads to lower pregnancy complications. However, this might be due to inadequate clinical and experimental studies.

Pregabalin (PGB) [S-(+)-3-isobutyl-GABA], as a new anticonvulsant drug is structurally related to gabapentin. However, it is a novel gamma-aminobutyric acid (GABA) analog which is virtually inactive at GABA receptors. It acts by binding to the α2δ-1 subunit of voltage dependent Ca^2+^ channels to reduce calcium currents through the cell membrane (9). It has many therapeutic applications and indications including treatment of central and peripheral neuropathic pain, adjunct therapy for partial seizures, generalized anxiety disorder, fibromyalgia and sleep disorders (10). PGB has been marketed after gabapentin and has the benefit of more efficacy and absorption. It has been reported that PGB can induce fetal structural abnormalities, lethality, growth retardation, and nervous system functional impairment and neural tube defect (NTD) at high doses (11). Also, few malformations have occurred when we investigated the teratogenic effect of gabapentin in mice (12).

Considering the broad therapeutic indications of PGB and scarcity of available information on its teratogenicity, we have investigated its potential teratogenic effects at lower doses. Also, due to the reported association of hyperhomocysteinemia with pregnancy complications and malformations such as heart defects and NTD caused by other AEDs (13, 14), we have measured the maternal serum homocysteine in PGB treated mice.

## Material and Methods

***Animal treatment***

PGB was purchased from Pfizer Inc, and alizarin red and alcian blue was provided from Merck (Germany). Eighty virgin female BALB/c mice, 10-12 weeks of age, body weight 20-30 g were used. They were obtained from Avicenna Research Institute of Mashhad University of Medical Sciences and were maintained under routine lighting conditions (12 hr light/dark cycles) and room temperature of (18-22°C) at least two weeks before the experiments. All animal experiments were approved by the Animal Care Committee of Mashhad University of Medical Sciences. Animals had free access to food and water until the evening before their euthanasia. Two females were caged with a male of the same strain overnight and the observation of a vaginal plug in the next morning was considered as gestational day (GD) zero .Three groups of pregnant mice (n=20) were intraperitoneally (IP) injected with PGB at doses of 20 (group I), 40 (group II) and 80 (group III) mg/kg/ day in two divided doses, during GD6–15 (organogenesis period). The control group received normal saline by the same route in an equivalent volume (n=20). 

***Maternal observation***

Maternal body weights were evaluated throughout pregnancy. All groups were observed daily for mortality, morbidity and general appearance. On the morning of GD18 the pregnant mice were euthanized. Their uterus were removed by Cesarean section, were weighed and the number of live and dead fetuses and resorption sites were recorded. Maternal body weight gain (MWG) was calculated by subtracting the weight of pregnant mice at GD0 from GD18. Then MWG minus the gravid uterine weight was obtained (MWGM). 

***Fetus observation and staining***

All fetuses were examined for external malformations, size (crown-rump length) and body weight. Microscopic observation of external malformations (exencephaly, cleft palate, abdominal hernia, polydactyl, open eyelid, etc.) was performed under a dissecting microscope. Malformed fetuses were then double stained with alizarin red and alcian blue according to Kimmel and Inouye and Trammel techniques (15). Skeletal anomalies were observed and recorded using a stereomicroscope. 

***Homocysteine measurement***

Blood samples were obtained by heart puncture from pregnant mice undergoing Caesarean section. After centrifugation, plasma samples were stored at -80°C until analysis. Total homocysteine level (tHcy) was measured by commercially available enzyme immunoassay (EIA) kit (Axis homocysteine EIA, axis-Shield Diagnostics Ltd, UK) according to the instructions of the manufacturer. Twenty five micro liter samples were required and the absorbance was measured at 405 nm using enzyme-linked immunosorbent assay (ELISA) reader (Statfax 2100, USA). The intra-assay coefficient of variation was <7%. The sensitivity of the assay was 2.0 mol/l. 

***Statistics***

Fetal body weight, crown-rump length and tHcy data are reported as mean±SEM. The unit of analysis for fetal body weight and crown-rump length was the litter mean and for tHcy was mol/l. Following ANOVA, Tukey test was done between control and each experimental group. Concerning the frequency of absorbed and live fetuses, external malformation differences between the control and each experimental group were tested with Fisher’s direct probability test and when the frequency of each category was 5 or more, the Chi-Square test was used. The statistical analysis was carried out with SPSS software (Ver. 17). *P*< 0.05 was considered significant.

## Results

***Maternal observation***

PGB administration resulted in a significant increase in fetal resorption frequency at 40 and 80 mg/kg/day. Treated groups were compared with the control group (*P* <0.05 and *P* <0.0001, respectively). The percentage of live fetuses also decreased at these doses (*P* <0.05 and *P* <0.0001, respectively). The highest incidence of resorption (56% vs. 17%) and the most decrease in the percentage of live fetuses (69.4% vs. 92.24%) occurred at the 80mg/kg dose. PGB did not cause significant changes in the number of implantations (Table1).

**Table 1:** Cesarean section parameters and external malformations in BALB/c mice fetuses exposed to *pregabalin*

Treatment and dose (mg/kg/day)				
	PGB (20) Group I	PGB (40) Group II	PGB(80) Group III	Control (Normal saline)
Dams (No)	20	20	20	20
Maternal weight gain, Mean ±SEM	18.03 ± 0.94	13.09 ± 0.73^ c^	11.46 ± 0.97^ c^	20.04 ± 1.01
MWGM, Mean ±SEM	4.58 ± 0.86	4.33 ± 0.35	2.72 ± 0.43	2.87 ± 0.67
Weight of uterus, Mean ±SEM	13.7 ± 0.74	10.37 ± 0.59^c^	8.59 ± 0.98^c^	15.46 ± 0.71
Number of implantation (Mean ±SEM)	186 (9.3 ± 0.5)	171(8.35 ± 0.8)	183 (9.15 ± 0.76)	219 (11.12 ± 0.61)
Number of live fetuses, No (%)	173 (93.01%)	139 (81.28%)^a^	127 (69.4%) ^c^	202 (92.24%)
Resorbed fetuses, No (%)	13(6.99%)	32 (18.71%)^a^	56 (30.60%) ^c^	17 (7.76%)
Fetal length, Mean ±SEM (mm)	21.48 ± 0.82	20.69 ± 0.58	21.78 ± 1.38	20.52 ± 0.67
Fetal weight, Mean ±SEM (g)	1.06 ± 0.06	1.08 ± 0.1	1 ± 0.06	1.17 ± 0.18
Severs malformation, No (%)	11(6.35%)^b^	7(5.03%) ^a^	3(2.36%)	2(0.1%)
Growth retardation,No (%)	5 (3.93%)^c^	1(0.72%)	1(0.58%)	0 (0%)

^a^ *P*<0.05, ^b ^*P*<0.01 and ^c ^*P*<0.001 compared to control group

Group I, II, III and control received 20, 40, 80 and 0 mg /kg/day of pregabalin, respectively

PGB: Pregabalin, MWGM: Maternal body weight gain (MWG) minus the gravid uterine weight

 As shown in Table 1, MWG was significantly decreased at the 40 and 80 mg/kg/day doses (*P*<0.001). The weight of uterus was also reduced at these doses significantly (*P*<0.001). However, MWGM did not change significantly. No mortality was detected in all groups.

***Fetus observation and staining***

**Figure 1:**
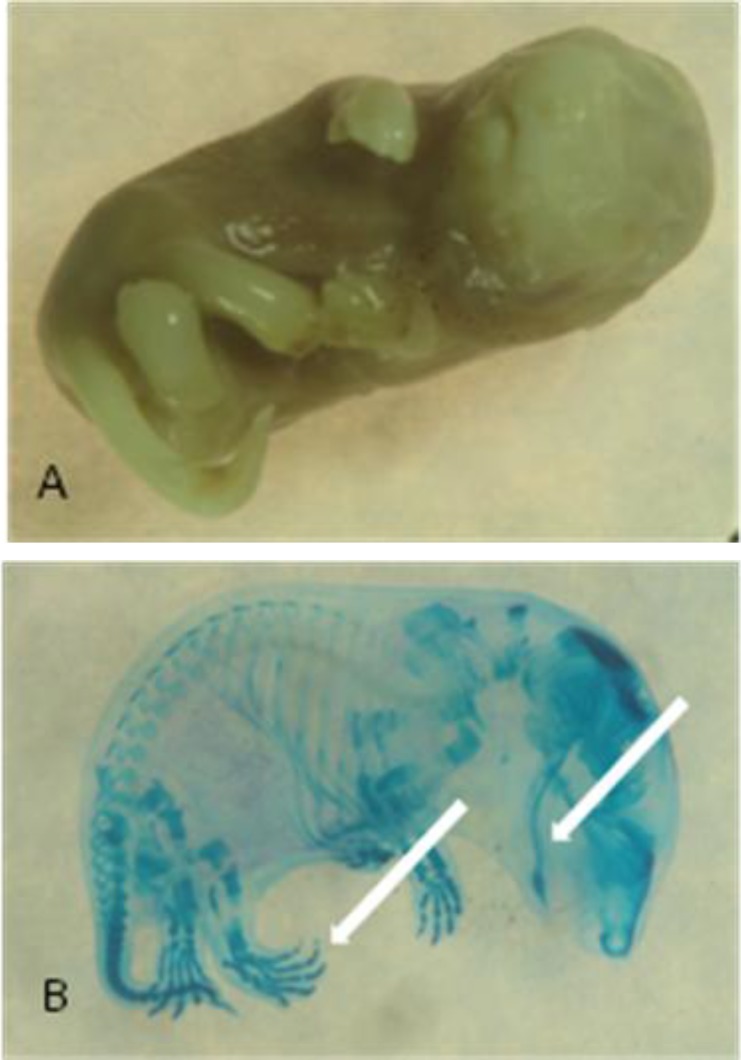
A fetus before (A) and after (B) skeletal staining with marked delay ossification, mandibular hypoplasia and clinodactyly (white arrow)from experimental group I, treated with 20 mg/kg/day pregabalin

**Figure2:**
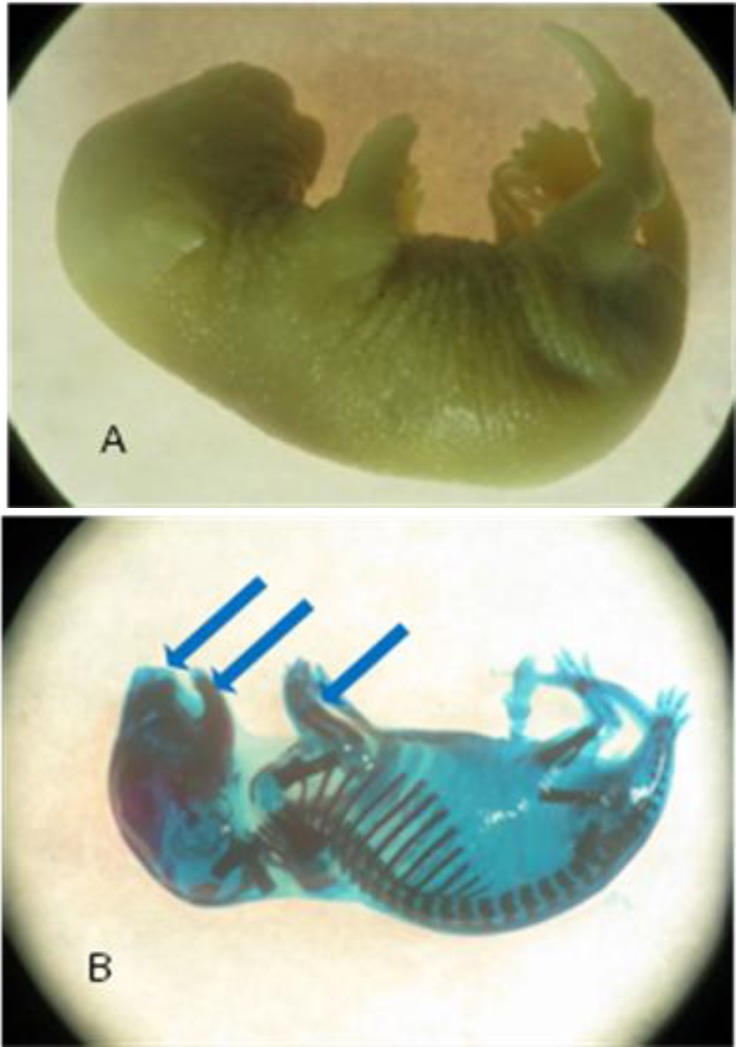
A fetus before (A) and after (B) skeletal staining with maxillary and mandibular deformities and disorder in ulna and radial formation (blue arrows) from experimental group I, treated with 20 mg/kg/day pregab

**Figure 3:**
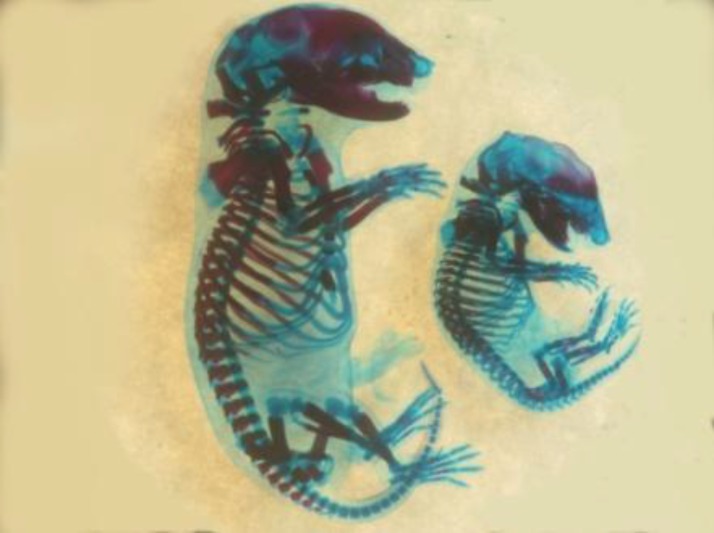
A fetus skeleton with scoliosis from experimental group I treated with 20 mg/kg/day pregabalin, which has been stained with Alizarin red S-Alcian blue. The right and left fetuses are malformed and non malformed, respectively

There was no significant difference between body weight and crown-rump length of the fetuses among four groups. The prevalence of gross malformations was significantly higher at groups I and II (*P*<0.05 and *P*<0.001, respectively). The rate of skeletal malformations of fetuses increased significantly at 20, 40 and 80 mg/kg/day doses when compared to the control group (*P*<0.001, *P*<0.05 and *P*<0.05, respectively, Table 2). The highest rate of skeletal abnormality was observed in the low dose group (20 mg/kg). The abnormalities included vertebral column deformity, limb deformity and craniofacial abnormalities. Limb deformity was the most prominent malformation that was observed with a higher incidence at group I (5.78% vs. 0%) (*P*<0.0001). These deformities included malrotation, delayed development in lower limbs and disorder in ulna and radial formation as well as clinodactyly (Figure 1, 2). Vertebral deformities were the second prevalent deformity determined as deviations in normal curvatures (Figure 3). Our results showed that %2.31 of fetuses in group I, 2.38% in group II and %1.57 in group III had this malformation (Table 2). Brachygnathia was the most prominent malformation (Figure 1 and 2). Although, there is not significant differences in craniofacial abnormalities between treated and control groups (Table 2).

**Figure 4:**
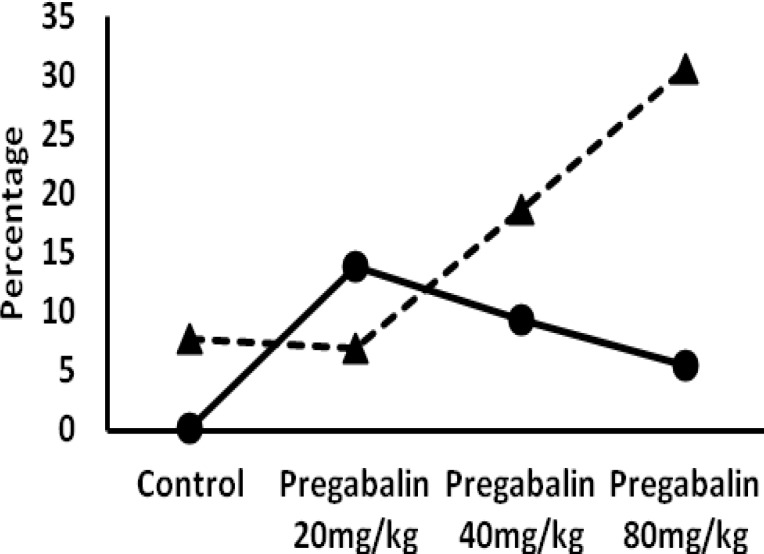
Comparison between percentages of pregnancy resulted in fetal resorption or malformation. The continuous and interrupted lines are related to percent of malformations and fetal resorptions, respectively, Chi-square: 29.305,* Df*= 6 (*P*<0.00001)

 It is noticeable that the percentage of pregnant mothers with fetal resorption or with malformation, increased significantly in all PGB administered test groups (*P*<0.0001) (Figure 4). It is noticeable that the percentage of pregnancy resulted in fetal resorption or malformation, increased significantly in all PGB administered test groups (*P*<0.0001) (Figure 4).

***Plasma homocysteine concentration***

**Figure 5:**
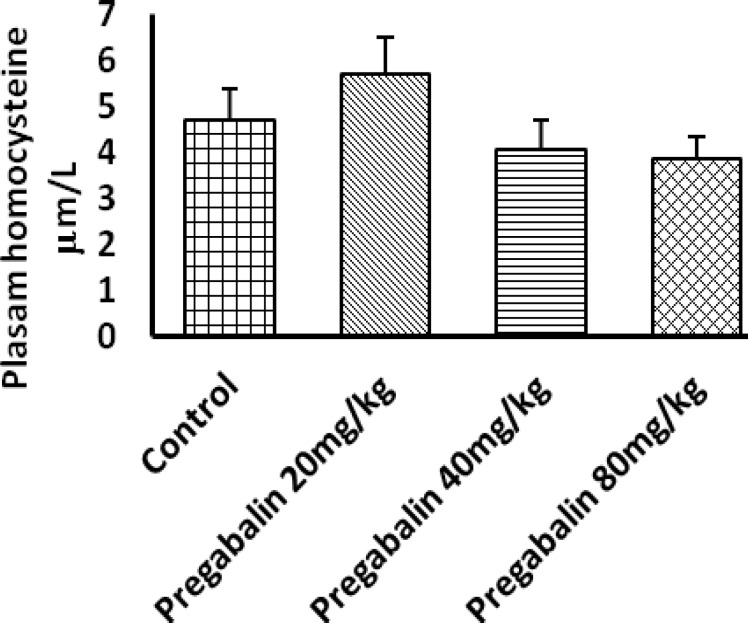
Homocysteine level at different doses. Serum tHcy levels in pregnant mice exposed to normal saline (control) or pregabalin at different doses, through 6-15 gestational days. Values are presented as mean±SEM. There was no significant difference between means of serum tHcy of different groups

There was no significant differences in tHcy between treated and control groups. We did not find any difference among treated groups either (Figure 5).

**Table 2:** Skeletal malformations in BALB/c mice fetuses exposed to *pregabalin*

	Treatment and dose (mg/kg/day)			
	PGB(20) Group I	PGB(40) Group II	PGB(80) Group III	Control (Normal saline)
Dams (No)	20	20	20	20
Fetuses examined	173	139	127	202
Vertebral column deformity, No (%)	4 (2.31%) ^a^	4 (2.88%)^ a^	2 (1.57%)	0 (0%)
Limb deformity, No (%)	10 (5.78%)^c^	3 (2.16%)	2 (1.57)	0 (0%)
Craniofacial abnormalities, No (%)	3 (1.73%)	1 (0.72%)	0 (0%)	0 (0%)
Total number of birth defect, No (%)	24 (13.87%)^b^	13 (9.35%)^a^	7 (5.51%) ^a^	2 (0.1%)

Group I, II, III and control received 20, 40, 80 and 0 mg /kg/day of pregabalin, respectively

PGB: Pregabalin

## Discussion

In a study to determine the proﬁle of PGB rodent model of epilepsy, PGB prevented behavioral and clonic seizure at 10 mg/kg (IP injection) and at 31 mg/kg (oral administration) in rat and mice, respectively. Since PGB does not bind to plasma proteins and has linear kinetic and bioavailability above 90%, the IP route of drug administration was chosen at doses that are much closer to the therapeutic range of PGB in animal experimentations (16). 

This study showed that PGB administration decreased MWG at doses of 40 and 80 mg/kg/day. However, means of MWGM were not significantly different. The reduction of MWG may be due to the increased prevalence of resorption sites at the higher doses. Increase of resorption sites resulted in a significant reduction of uterine weight compared to the control group at the mentioned doses. Therefore, it can be concluded that PGB treatment can not affect the weight gain of the mice during pregnancy. It is noteworthy that the results of randomized controlled trials indicated that PGB therapy can cause bodyweight gain and/or fluid retention. This was correlated with other anticonvulsants or other drugs that were consumed with PGB, although it has not been investigated on pregnant woman yet (17, 18).

According to the published information by the manufacturer, PGB can cause reduction in rat offspring growth at ≥ 100 mg/kg and decreased fetal body weight at >250 mg/kg in rabbit offspring (11). However PGB exposure in our study did not induce any change in body weight and crown-rump length of the fetuses. It may be due to differences in the doses and animal species.

Our study showed that PGB could cause fetal abnormalities in all treated groups compared to the control group. Schaefer and collaborators reported that exposure to PGB during the first trimester in a small sample of pregnant women could not induce substantial teratogenicity. On the other hand, high doses of PGB induced skeletal abnormalities and NTDs in some animal experiments (19). It was reported that PGB can be teratogenic in rat at higher doses of 1250-2500 mg/kg. Though, it was not teratogenic in mice or rabbits (20). Prakash *et al* reported that the IP injection of 113, 226, or 452 mg/kg doses of gabapentin, an ADE similar to PGB, at three different gestational stages induced fetal resorptions, growth retardation and various gross malformations in all treated groups at mid gestation (21). In our study, although the total frequency of birth defects was decreased, the prevalence of resorption sites was significantly raised through increasing the dose (in dose dependent manner). This means that at higher concentrations of PGB, more fatalities occurs early in embryonic development. Thus, the malformed embryos had been resorbed before showing any gross anatomical malformation. Hence, this would explain the lower “malformation rate” with increasing concentrations of PGB. Therefore, we suggest that PGB may cause embryotoxicity in a dose-dependent fashion as the excessive toxicity of the higher doses causes more resorptions and fetal mortalities.

 The results presented here have indicated that skeletal defects occur at doses as low as 20 mg/kg/day delivered IP. In our investigation, limb deformities were more frequent and mostly included malrotated limbs (Figure 1). The cellular mechanism of PGB effects is not very clear. However, it is claimed that the pharmacologic effects of PGB are related to its effect on the calcium channel α2–δ Type 1 subunit. This subunit is highly expressed in the skeletal, cardiac and vascular smooth muscles and in the brain (22). It is claimed that the absence of the α2–δ1 subunit in young myoblasts impairs migration, attachment and spreading of cells (23). Therefore, this protein function is crucial for muscle development and muscle repair. This provides a possible mechanism of limb deformity.

We identified scoliosis as the most common kind of vertebral deformities, as gabapentin induced (24) (Figure 3). Exposure to higher doses of PGB in animals (5-16 times of the maximum recommended dose in humans) caused a reduction in ossification rate, but not dose dependent (25). It may be another explanation for the skeletal deformity at different doses.

In this investigation, we could not detect any NTD. In our previous study, we have found that, mechanistically similar to PGB, gabapentin induced malformations categorized as skeletal malformation and NTD. However, in another study that we administrated gabapentin during the ﬁrst ten days of pregnancy the incidence of NTD was not significant. Skeletal malformations induced by gabapentin exposure mostly included malrotated limbs and micromelia (12). 

Hyperhomocysteinemia (HHcy) has been considered as a risk factor for some diseases and birth defects such as vascular disease, Alzheimer disease, osteoporosis and NTD (26-29). Also, significant correlation has been shown between HHcy and recurrent miscarriage in pregnant women (30). Also, it has been reported that some AEDs can cause HHcy (31). Regarding these information gabapentin-induced NTD (12) and nervous system functional modification induced by PGB (11), evaluation of HHcy was considered in our study. However, our results showed no significant difference in the tHcy level among all groups. It can indicate failure of correlation between this risk factor and PGB induced pregnancy complications.

## Conclusion

In summary, according to our findings PGB may have potential teratogenic effects even in lower doses though with less intensity than other AEDs. It can affect especially bone skeletal development. It seems that PGB can also increase the risk of miscarriage and decrease in normal pregnancy outcome. Therefore, it is suggested that great caution should be taken when prescribing PGB during pregnancy and further investigation for possible mechanisms should be performed.
